# KMT2D deficiency disturbs the proliferation and cell cycle activity of dental epithelial cell line (LS8) partially via Wnt signaling

**DOI:** 10.1042/BSR20211148

**Published:** 2021-11-18

**Authors:** Liping Pang, Hua Tian, Xuejun Gao, Weiping Wang, Xiaoyan Wang, Zhichun Zhang

**Affiliations:** 1 Department of Cariology and Endodontology, School and Hospital of Stomatology, Peking University, Beijing 100081, P.R. China; 2 Department of Biochemistry and Molecular Biology, School of Basic Medical Sciences, Peking University, Beijing 100191, P.R. China

**Keywords:** epithelial cell proliferation, Histone methylation, KMT2D, tooth development, Wnt

## Abstract

Lysine methyltransferase 2D (KMT2D), as one of the key histone methyltransferases responsible for histone 3 lysine 4 methylation (H3K4me), has been proved to be the main pathogenic gene of Kabuki syndrome disease. Kabuki patients with *KMT2D* mutation frequently present various dental abnormalities, including abnormal tooth number and crown morphology. However, the exact function of KMT2D in tooth development remains unclear. In this report, we systematically elucidate the expression pattern of KMT2D in early tooth development and outline the molecular mechanism of KMT2D in dental epithelial cell line. KMT2D and H3K4me mainly expressed in enamel organ and *Kmt2d* knockdown led to the reduction in cell proliferation activity and cell cycling activity in dental epithelial cell line (LS8). RNA-sequencing (RNA-seq) and Kyoto Encyclopedia of Genes and Genomes (KEGG) enrichment analysis screened out several important pathways affected by *Kmt2d* knockdown including Wnt signaling. Consistently, Top/Fop assay confirmed the reduction in Wnt signaling activity in *Kmt2d* knockdown cells. Nuclear translocation of β-catenin was significantly reduced by *Kmt2d* knockdown, while lithium chloride (LiCl) partially reversed this phenomenon. Moreover, LiCl partially reversed the decrease in cell proliferation activity and G_1_ arrest, and the down-regulation of Wnt-related genes in *Kmt2d* knockdown cells. In summary, the present study uncovered a pivotal role of histone methyltransferase KMT2D in dental epithelium proliferation and cell cycle homeostasis partially through regulating Wnt/β-catenin signaling. The findings are important for understanding the role of KMT2D and histone methylation in tooth development.

## Introduction

Tooth development starts from the formation of dental lamina, after which dental epithelial cells proliferate downward to form a tooth bud and then the enamel organ [1]. The stable control of dental epithelium proliferation is also essential for the formation of the enamel knot, which determines the morphology of the enamel organ and tooth crown. Studies on the mechanisms underlying dental epithelial cell proliferation are crucial for understanding tooth development, which would help to lay a foundation for tooth regeneration. Classical signaling pathways have been widely found to take part in dental epithelium proliferation and tooth development [2–4]. For example, Wnt/β-catenin signaling is considered to play crucial roles at different stages of tooth formation [5,6]. Studies have shown that the formation and early morphogenesis of mouse teeth were arrested when canonical Wnt signaling was inhibited by DKK1 or loss-of-function of β-catenin or Lef1 [5,7,8]. Recently, the importance of epigenetic regulation has been gradually emphasized in embryonic development, since that the possible reversible ability of epigenetic messages such as histone modification showed great therapeutic potential [9–11]. Whereas, the key epigenetic factors and underlying mechanisms in tooth development remain to be determined, especially in dental epithelial tissues [12–14].

The lysine methyltransferase 2D (KMT2D) protein is an important histone methyltransferase, whose encoding gene has been identified as the main causative factor of Kabuki syndrome (OMIM #147920) [15,16]. Patients with Kabuki syndrome are characterized by a peculiar face, postnatal growth retardation and dental anomalies. Dental anomalies in Kabuki syndrome include congenital absence of teeth, short crowns, spiral or conical incisors and abnormal cusp morphology of molars [17–19]. The anomalies of tooth number and crown morphology in Kabuki syndrome suggest that KMT2D might function in the formation and morphogenesis of dental enamel organ. Morpholino-mediated *kmt2d* knockdown zebrafish exhibited significant craniofacial defects, including complete loss of three to seven pharyngeal arches [16]. Porntaveetus et al*.* reported specific mRNA expression of *KMT2D* in human dental epithelium during the bud and cap stages by *in-situ* hybridization [19]. These findings suggested that KMT2D might act as an important epigenetic factor in craniofacial and dental epithelial tissues. A further study of KMT2D function would be beneficial for understanding the epigenetic regulation during tooth development and regeneration.

Functionally, KMT2D is mainly responsible for histone 3 lysine 4 monomethylation (H3K4me1) or trimethylation (H3K4me3), which modifies enhancers or promoters for transcription factor binding and gene activation [20,21]. KMT2D plays important roles in the regulation of different classical signaling pathways in embryogenesis and tissue development. In human epidermal keratinocytes, knockdown of KMT2D resulted in inhibition of cell proliferation by altering the H3K4me1 modification on the enhancer of p63 [22]. During tooth development, several histone modifying enzymes such as KDM2A and EZH2, were reported to play essential roles in dental mesenchyme proliferation and differentiation [23–25]. However, in dental epithelial tissue, the functions and mechanisms of histone modification enzymes including KMT2D were unclear. In the present study, we systematically studied the expression pattern of KMT2D during early tooth development, and further investigated the specific role and mechanism of KMT2D in dental epithelial cell line (LS8).

## Materials and methods

### Tissue specimens and immunofluorescence assay

The present study was carrie d out in strict accordance with the National Institutes of Health Guide for the Care and Use of Laboratory Animals (National Resource Council). All protocols were approved by the Animal Care and Use Committee of Peking University (permit number: LA2017136). Pregnant ICR mice (Charles River, China) were killed by cervical dislocation after anesthesia with 5 mg/100 g body weight of sodium pentobarbital in the postprandial state. All the animal work took place in School and Hospital of Stomatology, Peking University.

Embryo heads were dissected and prepared as frozen specimens. Specimens were fixed in 4% paraformaldehyde at 4°C and then dehydrated with a gradient sucrose solution. The frozen specimens were cryosliced into 5-µm-frozen sections (CM1900, Leica, Germany). The sections were perforated with Triton X-100, blocked with goat serum, and then incubated with anti-KMT2D (C-Term, a smaller C-terminus fragment which separates at ∼75 kDa, ABE206, Sigma–Aldrich, U.S.A.), anti-H3K4me1 (GTX54100, GeneTex, U.S.A.), anti-H3K4me3 (GTX128954, GeneTex), anti-Ki67 (ab16667, Abcam, U.S.A.) and anti-phospho-Rb (S780) (YP0240, Immunoway, U.S.A.) separately at 4°C overnight. Then, the sections were incubated with fluorescein-conjugated rabbit anti-goat IgG (H+L) (ZF-0314, ZSGB-BIO, China) and anti-fluorescence quenching sealing tablets with DAPI (ZLI-9557, ZSGB-BIO, China). Fluorescence images were acquired by confocal laser microscopy (LMS710, Zeiss, Germany).

### Construction of LV-KMT2D-shRNA vector system and lentivirus transfection

The shRNA was purchased from GenePharma (Shanghai, China). The shRNAs were ligated into the pGLV3/GFP-puro vector with a constitutive CMV promoter to produce pGLV3-*Kmt2d*-shRNA. The lentivirus vector encodes green fluorescent protein (GFP), allowing rapid visual assessment of the viral infection efficiency. The recombinant pGLV3-GFP-*Kmt2d*-shRNA vector was termed sh*Kmt2d*. A negative control vector (pGLV3-GFP-NC-shRNA, termed ‘scramble’) with a scramble shRNA insert was used to control any effects caused by non-RNAi mechanisms. The shRNA sequences are as follows: *Kmt2d*-shRNA (5′-GGGAGTATCCACGGATGTTAG-3′); Scramble shRNA (5′-TTCTCCGAACGTGTCACGT-3′).

The LS8 mouse ameloblast cell line was kindly provided by Dr. Malcolm L. Snead (University of Southern California). For lentivirus transfection, cells at 60% confluence were transfected with lentivirus (MOI = 40) in Opti-MEM (Gibco, U.S.A.) containing 5 µg/ml polybrene for 24 h. Cells were selected on 2% puromycin after infection to obtain complete puromycin-resistant cell populations. GFP expression in cells was observed under a fluorescence microscope (IX53; Olympus, Japan) to determine the transfection efficiency. RNA was harvested at 48 h to detect knockdown efficiency.

### Quantitative reverse transcription polymerase chain reaction

Total RNA was isolated using TRIzol reagent (Invitrogen, U.S.A.) according to the manufacturer’s instructions. cDNA was synthetized in a 20-μl reaction mixture containing 1 μg total RNA, 4 μl of 5× Master Mix (Takara Bio Inc, Japan) that includes all reagents required for cDNA synthesis (PrimeScript RTase, RNase inhibitor, random 6-mers, oligo dT primer, dNTPs and reaction buffer) and RNA-free water. PCR was running in an ABI Q3 Real-Time PCR System (ABI, Singapore) with SYBR Green Master Mix (Roche, Switzerland) and the appropriate primers (Table 1). The annealing temperature was 61°C. Target transcript levels were normalized to that of *Gapdh*, and each value was the average of three independent experiments.

**Table 1:** A detailed list of primer sequences, species, GeneBank numbers and PCR product lengths used in real-time RT-PCR

Gene	Primer sequence	GeneBank accession number	PCR product size (bp)
*KMT2D*	S 5′-CCTGAGGAACCAAGCCAGAG-3′	NM_001033276.3	237
	AS 5′-TGCAGCTGGGATAATGGGTG-3′		
*CyclinD1*	S 5′-GCGTACCCTGACACCAATCTC-3′	NM_007631	183
	AS 5′-CTCCTCTTCGCACTTCTGCTC-3′		
*CDK4*	S 5′-ATGGCTGCCACTCGATATGAA-3′	NM_009870	129
	AS 5′-TCCTCCATTAGGAACTCTCACAC-3′		
*CyclinB1*	S 5′-AAGGTGCCTGTGTGTGAACC-3′	NM_172301	228
	AS 5′-GTCAGCCCCATCATCTGCG-3′		
*CyclinB2*	S 5′-GCCAAGAGCCATGTGACTATC-3′	NM_007630	114
	AS 5′-CAGAGCTGGTACTTTGGTGTTC-3′		
*CDK1*	S 5′-AGAAGGTACTTACGGTGTGGT-3′	NM_007659	128
	AS 5′-GAGAGATTTCCCGAATTGCAGT-3′		
*Lgr4*	S 5′-CCCGACTTCGCATTCACCAA-3′	NM_172671	152
	AS 5′-GCCTGAGGAAATTCATCCAAGTT-3′		
*Wnt9a*	S 5′-GGCCCAAGCACACTACAAG-3′	NM_139298	238
	AS 5′-AGAAGAGATGGCGTAGAGGAAA-3′		
*Wnt10b*	S 5′-GAAGGGTAGTGGTGAGCAAGA-3′	NM_011718	158
	AS 5′-GGTTACAGCCACCCCATTCC-3′		
*Tle2*	S 5′-TGGCTGCCGTAAAGGAAGAC-3′	NM_001251401	191
	AS 5′-CTCACTGTCATAAGGCCCTGA-3′		
*Tle6*	S 5′-ATCCAGTCGGTATTTGTCCATCG-3′	NM_053254	147
	AS 5′-AGGTCTGGGGTTCTACTGAAG-3′		
*TCF4*	S 5′-CGAAAAGTTCCTCCGGGTTTG-3′	NM_013685	196
	AS 5′-CGTAGCCGGGCTGATTCAT-3′		
*Axcin2*	S 5′-TGACTCTCCTTCCAGATCCCA-3′	NM_015732	105
	AS 5′-TGCCCACACTAGGCTGACA-3′		
*β-catenin*	S 5′-ATGGAGCCGGACAGAAAAGC-3′	NM_007614.3	108
	AS 5′-CTTGCCACTCAGGGAAGGA-3′		
*Lef1*	S 5′-TGTTTATCCCATCACGGGTGG-3′	NM_010703	67
	AS 5′-CATGGAAGTGTCGCCTGACAG-3′		
*p21*	S 5′-CCTGGTGATGTCCGACCTG-3′	NM_001111099	103
	AS 5′-CCATGAGCGCATCGCAATC-3′		
*Bmi1*	S 5′-ATCCCCACTTAATGTGTGTCCT-3′	NM_007552	116
	AS 5′-CTTGCTGGTCTCCAAGTAACG-3′		
*Gli1*	S 5′-CCAAGCCAACTTTATGTCAGGG-3′	NM_010296	130
	AS 5′-AGCCCGCTTCTTTGTTAATTTGA-3′		
*Ptch1*	S 5′-AAAGAACTGCGGCAAGTTTTTG-3′	NM_008957	164
	AS 5′-CTTCTCCTATCTTCTGACGGGT-3′		
*Lfng*	S 5′-CGAGGTGCATAGCCTCTCC-3′	NM_008494	133
	AS 5′-GCGAGGGGACAGAACTTCG-3′		
*GAPDH*	S 5′-CCAGCCTCGTCCCGTAGACA-3′	NM_008084	189
	AS 5′-CCGTTGAATTTGCCGTGAGT-3′		

### Western blotting

Total protein was extracted with RIPA lysis buffer. Nuclear protein was extracted using the Nuclear-Cytosol Extraction kit (Applygen Technologies, Beijing, China). Equal amount of denatured proteins (30 µg) were separated by 12% sodium dodecyl sulfonate/polyacrylamide gel electrophoresis and transferred on to a polyvinylidene difluoride membrane. The membrane was incubated in 5% dry skim milk at room temperature (RT) for 1 h, and subsequently with anti-KMT2D (C-Term, a smaller C-terminus fragment which separates at ∼75 kDa, 1:1000, ABE206; Millipore, U.S.A.), anti-β-catenin (1:1000, 8480S; CST, U.S.A.), anti-H3K4me1 (1:1000, GTX54199; GeneTex), anti-H3K4me3 (dilution 1:1000, GTX128954; GeneTex), anti-Lef1 (dilution 1:1000, 2230S; Cell Signaling Technology), anti-Lgr4 (1:1000, sc-390630; Santa Cruz, U.S.A. ), GAPDH (1:10000, 10494-1-AP; Proteintech, China) and anti-LaminB1 (1:5000, 66095-1-Ig; Proteintech) separately at 4°C overnight. Antibody recognition was detected with horseradish peroxidase-coupled goat anti-rabbit IgG (1:5000, ZB-5301; ZSGB-BIO, China) or goat anti-mouse IgG (1:5000, ZB-5305; ZSGB-BIO, China) for 1 h at RT. GAPDH was used as an internal control for total protein detection, and LaminB1 for nuclear protein. The presentative images of three independent experiments were presented.

### Cell proliferation assay (CCK8) and colony formation assay

For the CCK8 cell proliferation assay, cells were seeded in 96-well plates at 2 × 10^3^ cells per well and starved for 12 h. Then, cells were incubated in 100-µl DMEM containing 10 µl of CCK8 solution (Dojindo, Japan) per well for 3 h on days 1, 2, 3, 4, 5 and 6. The optical density at 450 nm in each well was measured using a microplate reader (BIO-TEK, U.S.A.). For cell colony formation assay, cells were seeded in six-well plates at 1 × 10^3^ cells per well and starved for 12 h, then cultured for 7 days. After fixing with 4% paraformaldehyde for 20 min, the cells were washed twice and incubated at RT for 10–15 min in Giemsa solution (Solarbio, Beijing, China). The Giemsa solution was discarded, followed by washing in PBS. The results of colony staining were recorded by an experimental scanner (G4050, HP, U.S.A.). The presentative images of three independent experiments were presented.

### EdU incorporation assay

For EdU incorporation assays, LS8 cells were uniformly seeded in 96-well plates at a density at 3 × 10^3^ cells per well and cultured in complete medium overnight. Then, the medium was replaced with DMEM containing 50 μM EdU (C10310-1, EdU Apollo 567 In Vitro Imaging Kit; Ribobio, China), and the cells were cultured for another 2 h. The cells were fixed in 3.7% formaldehyde in PBS at RT for 15 min, incubated in 2 mg/ml glycine and treated with 0.5% Triton in PBS twice for 10 min, and then incubated with 1× Apollo® staining liquid in the dark at RT for 30 min. Coupling of EdU to the Apollo staining liquid substrate was observed under a fluorescence microscope (IX53; Olympus, Japan). The presentative images of three independent experiments were presented.

### Cell cycle analysis

LS8 cells were digested with 0.25% trypsin-EDTA, washed twice with cold PBS and fixed with 70% ethanol at 4°C overnight. The cells were washed twice with ice-cold PBS and then incubated in 0.5 ml of PI/RNase Staining Buffer (BD Pharmingen, U.S.A.) for 15 min in the dark. DNA content was measured using a flow cytometer (Epics XL; Beckman Coulter, U.S.A.) within 60 min. The experiment was performed at least three times.

### RNA-sequencing

RNA was extracted from LS8 cells stably transfected with *Kmt2d* shRNA and scramble shRNA (three biological replicates each) and was sent out for RNA library construction and sequencing at the Beijing Genomics Institution (Shenzhen, China). Total RNA quality and cDNA library quality were tested on an Agilent Bioanalyzer 2100. RNA-sequencing (RNA-seq) was conducted on the BGISEQ-500 sequencing platform. The raw sequencing reads were filtered to obtain clean data without reads with adaptors and low-quality reads. The clean reads were mapped to reference genes using Bowtie2-mm10 and to a reference genome using HISAT-mm10. RSEM was used to calculate transcript expression levels. Subsequently, the DEGseq algorithm was used for differential gene detection. Finally, differential expression genes (DEGs) were screened and annotation analysis of Gene Ontology (GO) was performed. Kyoto Encyclopedia of Genes and Genomes (KEGG) pathway enrichment analyses based DEGs database through statistical tests to identify the differential gene involved in the cell proliferation pathway and tooth development pathways.

### Cell immunofluorescence assay

LS8 cells were uniformly seeded on slides at 1 × 10^5^ cells per well, cultured in high-glucose DMEM (Gibco, U.S.A.) overnight, and then cultured in serum-free DMEM for 12 h. The starved cells were treated with lithium chloride (LiCl; 5 mM) for 24 h. The cells were fixed with 4% paraformaldehyde for 15 min and then treated with 0.1% Triton X-100 for 30 min and immersed in blocking agent at RT for 1 h. The cells were incubated with anti-β-catenin antibody (1/300 dilution, 8480S; Cell Signaling Technology, U.S.A.) at 4°C overnight, and then with fluorescein-conjugated rabbit anti-goat IgG (H+L) (1/200; ZSGB-BIO). Nuclei were counterstained using anti-fluorescence quenching sealing tablets with DAPI (1/200; ZSGB-BIO). Fluorescence images were acquired by confocal laser microscopy (LMS710). All slides were processed at the same time to ensure homogeneity of the staining procedures.

### Wnt/β-catenin signal reporter assay

Stably transfected LS8 cells were plated at a concentration of 5 × 10^5^ cells per well in 12-well plates. Cells were transfected with 0.5 μg TOP-flash or FOP-flash expression plasmids and 0.05 μg pRL-TK (Upstate Technology, U.S.A.) as control using Lipofectamine® 2000 (Invitrogen, U.S.A.). Luciferase activity was measured using a Dual-Luciferase® Reporter Assay System (Beyotime, China) and Microporous plate luminometer (LB-960; Berthold, U.S.A.) in accordance with the manufacturers’ instructions. The TOP/FOP ratio was used as a measure of TCF/LEF transcriptional activity. The experiment was performed three times.

### Statistical analysis

All data were presented as the mean ± SD. Means were compared using Student’s *t* test between two groups. A normality test was run before one-way ANOVA when comparing three groups or more. Prism 8.0.2 statistical software (GraphPad, U.S.A.) was used for statistical analysis. *P*<0.05 was considered significant.

## Results

### Spatiotemporal pattern of KMT2D and H3K4me1/3 expression during mouse molar development

**Figure 1:**
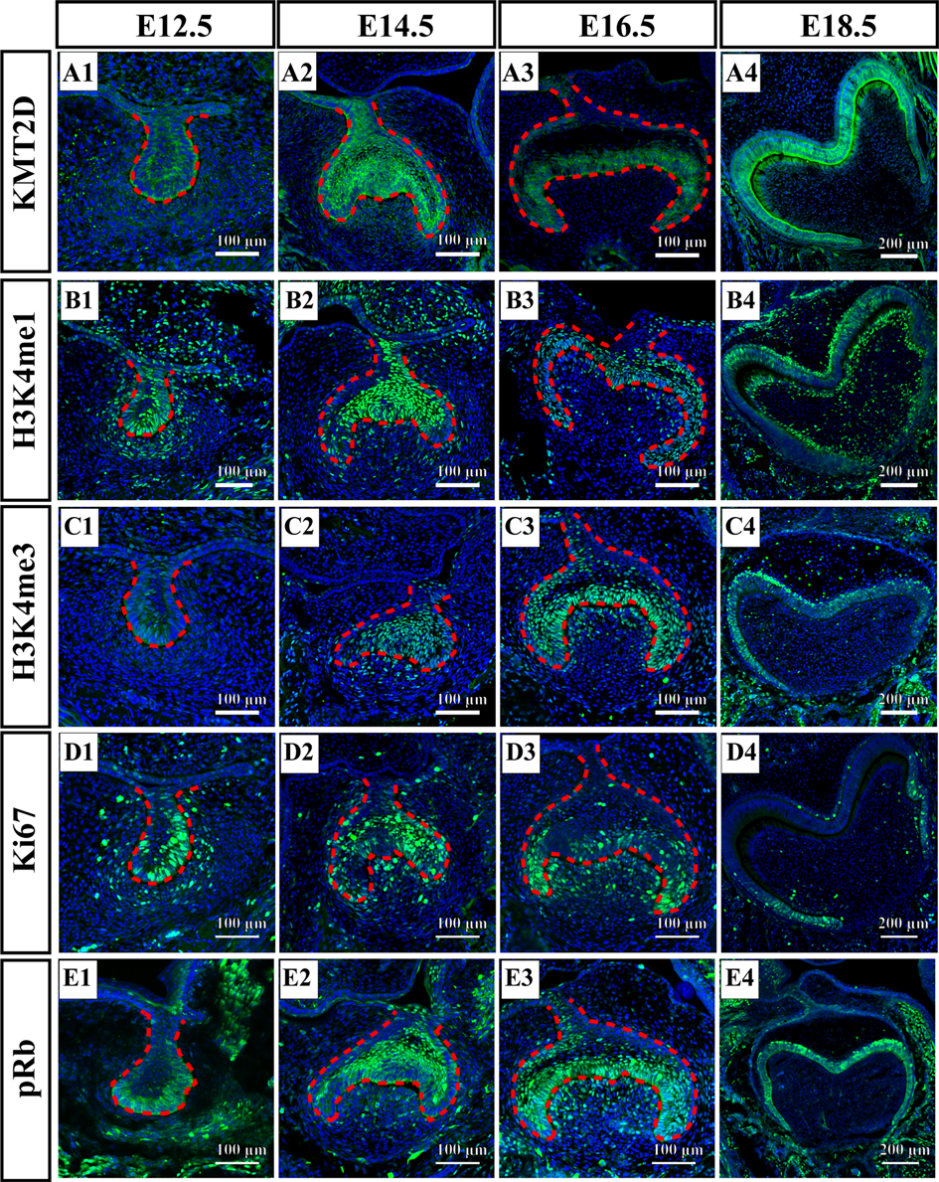
Spatiotemporal pattern of KMT2D, H3K4me1/3, Ki67 and pRb during early tooth germ development (**A1**–**A4**) showed the expression of KMT2D in tooth germ at E12.5 (bud stage), E14.5 (cap stage), E16.5 (early bell stage), E18.5 (late bell stage), respectively. (**B1**–**B4**) showed the expression of H3K4me1 in tooth germ at E12.5, E14.5, E16.5, E18.5. (**C1**–**C4**) showed the expression of H3K4me3 in tooth germ at E12.5, E14.5, E16.5, E18.5. (**D1**–**D4**) showed the expression of Ki67 in tooth germ at E12.5, E14.5, E16.5, E18.5. (**E1**–**E4**) showed the expression of pRb in tooth germ at E12.5, E14.5, E16.5, E18.5. The enamel organ was labeled by red-dotted line. The data represent three independent experiments, each in triplicate. Scale bar: 100 µm (E12.5, E14.5, E16.5); 200 µm (E18.5). Abbreviation: pRb, phosphorylation retinoblastoma protein, Ser^780^.

The expression pattern of KMT2D protein during mouse molar development was detected using immunofluorescence staining. The results showed that during the bud stage (Embryonic Day 12.5, E12.5, Figure 1A1) and cap stage (Embryonic Day 14.5, E14.5, Figure 1A2) of mouse tooth germs, KMT2D was expressed mainly in dental epithelium, whereas few signals were observed in the mesenchyme. At the early bell stage (Embryonic Day 16.5, E16.5, Figure 1A3), KMT2D was specifically distributed in the inner enamel epithelium, with no signal observed in the dental papilla. At the late bell stage (Embryonic Day 18.5, E18.5, Figure 1A4), KMT2D signals were observed in both pre-ameloblasts and pre-odontoblasts.

Consistent with KMT2D, H3K4me1 signals were observed in dental epithelium from bud to early bell stages (E12.5, E14.5, E16.5), and H3K4me1 signals were much stronger than that of KMT2D in the dental mesenchyme (Figure 1B1–B3). At the late bell stage (E18.5), H3K4me1 was dispersed in pre-ameloblasts, pre-odontoblasts and stellate reticulum cells (Figure 1B4). H3K4me3 signals were mainly distributed in the dental epithelial cells at all stages, with few signals observed in dental mesenchymal cells at the cap and early bell stages (Figure 1C1–C4).

Additionally, we detected Ki67 and pRb (phosphorylation retinoblastoma protein, Ser^780^) in different stages of mouse molar development. Ki67, a cell proliferation marker, was marked by green fluorescence (Figure 1D1–D4) in dental epithelium and mesenchyme. pRb (Ser^780^), a crucial mediator in mediating cell cycle G_1_/S transition, was detected during the tooth germ development (Figure 1E1–E4). The expression patterns of KMT2D, Ki67 and pRb (Ser^780^) revealed similarity from the bud to early bell stages, especially in the dental epithelial region. At the late bell stage (E18.5), pRb expression regions were still similar with KMT2D and H3K4me1/3, while Ki67 was expressed only in regions near the cervical loop.

### KMT2D knockdown resulted in reduction in proliferation activity and H3K4me1/3 expression

**Figure 2:**
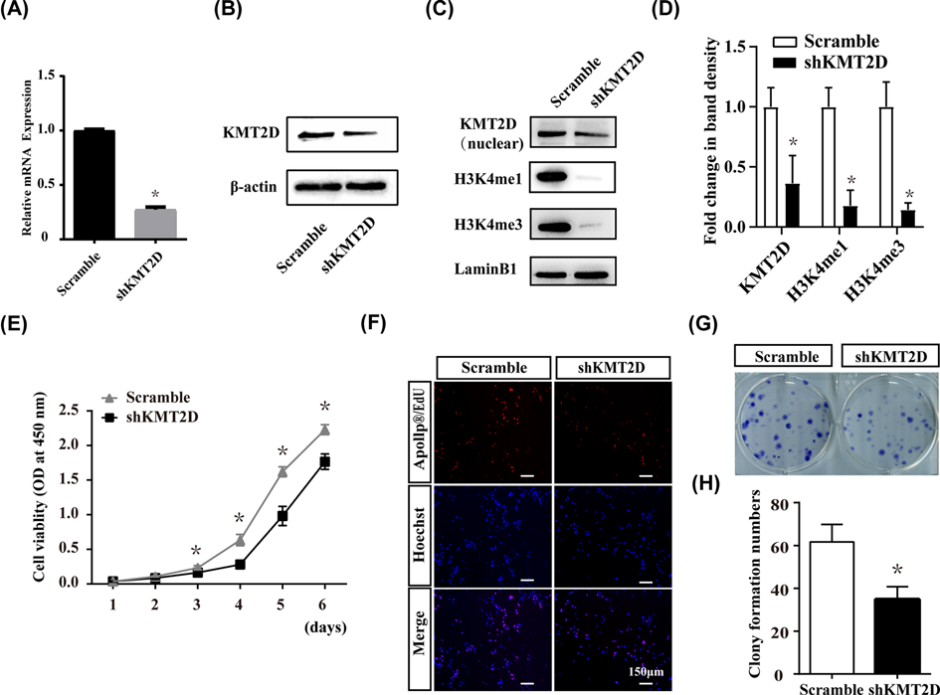
Knockdown of KMT2D resulted in the attenuation of proliferation ability and decreased H3K4me1/3 level of LS8 cells The knockdown efficiency of KMT2D was confirmed by real-time RT-PCR (**A**) and Western blot (**B**). The protein levels of nuclear H3K4me1 and H3K4me3 were decreased in shKMT2D group (**C**). Quantitative data of nuclear H3K4me1 and H3K4me3 protein by Western blot (**D**). Proliferation of LS8 cell lines was measured by CCK8 assay (**E**). OD values at 450 nm spectrum of three independent experiments were presented. (**F**) Result of EdU incorporation assays showed active DNA replication during cell proliferation (red dots). Representative results (from triplicate wells) of three experiments was shown. (**G**) Cell propagation was measured by the colony formation assay. Representative result (from triplicate wells) of three experiments was shown. (**H**) Quantitative data of the colony formation assay of three independent experiments were presented. The data represent three independent experiments, each in triplicate. Each bar represents mean ± SD from three samples (*P<0.05). Scramble: LS8 cells transfected with Scramble shRNA. shKMT2D: LS8 cells transfected with KMT2D shRNA. Means were compared using Student’s *t* test.

To investigate the effect of KMT2D on dental epithelial cells, we established a stable KMT2D knockdown cell model using lentivirus transduction. Transfection efficiency of shKMT2D was detected at both mRNA and protein levels (Figure 2A,B). Meanwhile, KMT2D knockdown caused significant decreases in global H3K4me1 and H3K4me3 levels (Figure 2C,D).

Cell proliferation activity was significantly decreased in KMT2D knockdown cells, as indicated by CCK8 assay (Figure 2E). EdU incorporation assay showed that substantially less EdU-positive cells were observed in the shKMT2D group than in the scramble group (Figure 2F), which was consistent with the CCK8 result. Colony formation assays revealed that the colony formation ability of shKMT2D cells was compromised, as evidenced by fewer colonies over the course of 10 days than that observed in scramble group (Figure 2G,H).

### KMT2D deficiency induced G_1_ phase arrest and down-regulation of cyclinD1 and cyclin-dependent kinase 1

**Figure 3:**
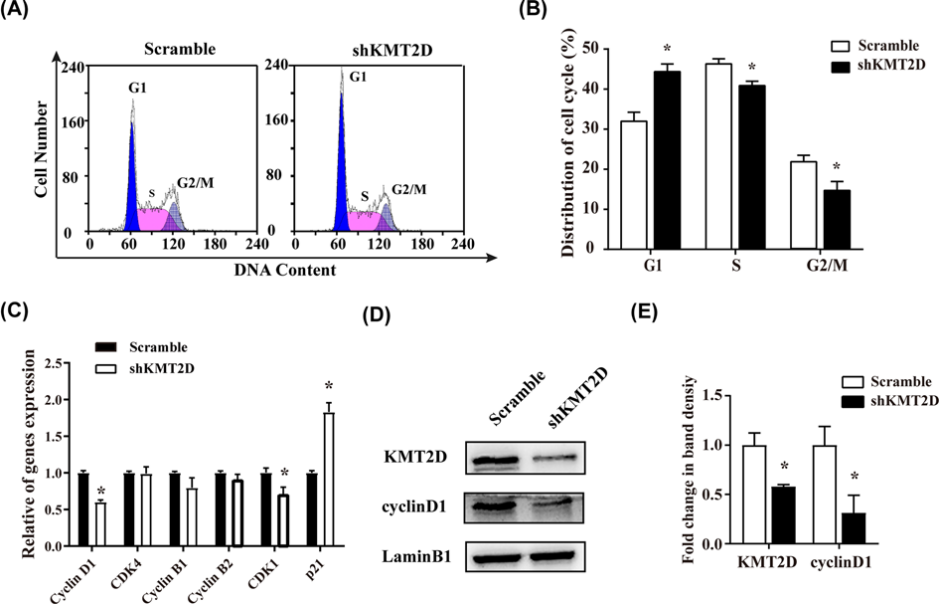
KMT2D deficiency induced reduction in cell cycling activity and a down-regulation of CyclinD1 and Cdk1 in LS8 cells (**A**) Representative cell cycle images measured using FCM. G_1_, S and G_2_/M phase were marked respectively in the images. (**B**) Quantitative data of cell cycle distribution showed G_1_ phase arrested. (**C**) The mRNA expression of cell cycle related genes (*CyclinD1*, *Cdk4*, *CyclinB1*, *CyclinB2* and *Cdk1*) were measured by real-time RT-PCR. (**D**) The protein level of nuclear KMT2D and CyclinD1 were detected by Western blot. (**E**) Quantitative data of nuclear CyclinD1 of three independent experiments were presented. The data represent three independent experiments, each in triplicate. Each bar represents mean ± SD from three samples (*P<0.05). Means were compared using Student’s *t* test.

The effects of KMT2D deficiency on cell cycle distribution were analyzed by flow cytometry. Compared with the scramble group, cells in the S and G_2_/M phases were significantly decreased in the shKMT2D group, suggesting that the cell cycle was blocked at the G_1_ checkpoint upon KMT2D knockdown (Figure 3A,B). The expressions of several key cell cycle regulatory proteins were then detected by quantitative reverse transcription polymerase chain reaction (RT-qPCR) and Western blotting. KMT2D deficiency led to a decrease in mRNA expression of *CyclinD1* and cyclin-dependent kinase (Cdk) 1 (*Cdk1*), which function in G_1_/S cell cycle transition. The mRNA expression of *p21*, a Cdk inhibitor, was obviously up-regulated, whereas other cell cycle-related factors, including *CyclinB1*, *CyclinB2* and *Cdk4* were not observably affected (Figure 3C). The expression of nuclear CyclinD1 protein was significantly down-regulated (Figure 3D,E).

### RNA-seq results revealed the effects of KMT2D deficiency on genes expression and key signaling pathways

**Figure 4:**
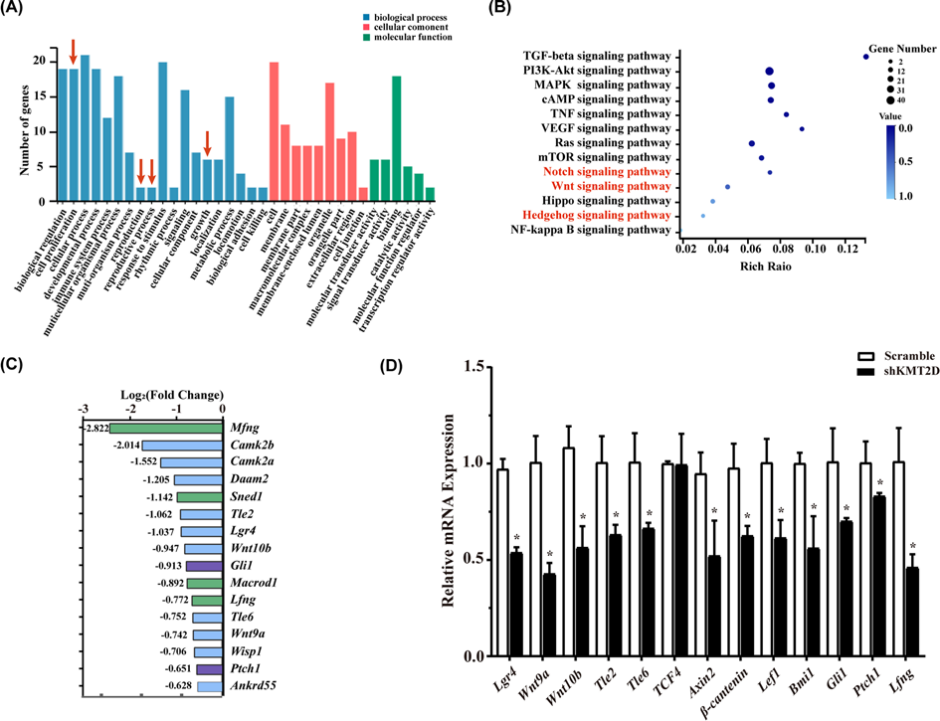
Bioinformatics analysis of GO term and KEGG pathway revealed the effects of KMT2D deficiency on cell function and signaling pathways There were 225 down-regulated genes (FC ≤ −2, Q value ≤ 0.05) and 99 up-regulated genes (FC ≥ 2, Q value ≤ 0.05). (**A**) GO analysis result displayed functional clustering of DEGs. The red arrows marked the functions such as cell proliferation, reproduction and growth. (**B**) Interested KEGG pathway results were presented by bubble diagram. (**C**) Genes related to Wnt (blue column), Notch (green column) and Hedgehog (purple column) signal pathway in the DEGs data were listed as log2 (fold of change). (**D**) Expression of above filtered genes were confirmed by real-time RT-PCR. Each bar represents mean ± SD from three samples (*P<0.05). Means were compared using Student’s *t* test.

The shKMT2D and scramble shRNA-transfected LS8 cells were subjected to RNA-seq. In general, DEGs were obtained, followed by the analysis of gene functions and signaling pathway. GO analysis of the filtered data revealed that genes related to numerous cell behavioral processes, including cell proliferation, reproduction and adhesion, were reduced after KMT2D knockdown (Figure 4A). KEGG pathway analysis revealed 307 signal pathways that were involved in the biological effects induced by KMT2D knockdown. There were 225 down-regulated genes (fold change (FC) ≤ −2, Q value ≤ 0.05) and 99 up-regulated genes (FC ≥ 2, Q-value ≤ 0.05). Based on the regulation of KMT2D on the proliferation of dental epithelial cell line, KEGG analysis results showed the signaling pathways that regulated cell proliferation and/or tooth development were represented in Figure 4B with a bubble diagram. The size and chroma of bubbles showed the genes number and the Rich Ratio, respectively. Among these signaling pathways, Wnt, Notch1 and Hedgehog pathways are extremely important pathways during tooth development, while PI3K and MAPK signaling pathways rarely plays a key role in tooth development, although a relatively large number of downstream genes in the PI3K and MAPK signaling pathway were affected by KMT2D knockdown. Given the important roles of the Wnt, Notch1 and Hedgehog pathways (highlighted in red font in Figure 4C) in early tooth development, all affected genes in these pathways were verified by RT-qPCR. Consistent with the RNA-seq data, the mRNA levels of *Lgr4, Wnt9a, Wnt10b, Tle2, Tle6, Axin2, Lef1* and *β-catenin* that involved in Wnt signaling were significantly decreased (Figure 4D). Notch pathway factor *Lfng* and Hedgehog pathway factor *Gli1* and *Ptch1* were also significantly decreased. The mRNA expression of *Bmi1*, a member of the polycomb protein family that acts as a Wnt signaling pathway activator, was also down-regulated by KMT2D knockdown.

### Activation of Wnt signaling by LiCl partially reversed the effects of KMT2D deficiency on cell proliferation and cell cycle

**Figure 5:**
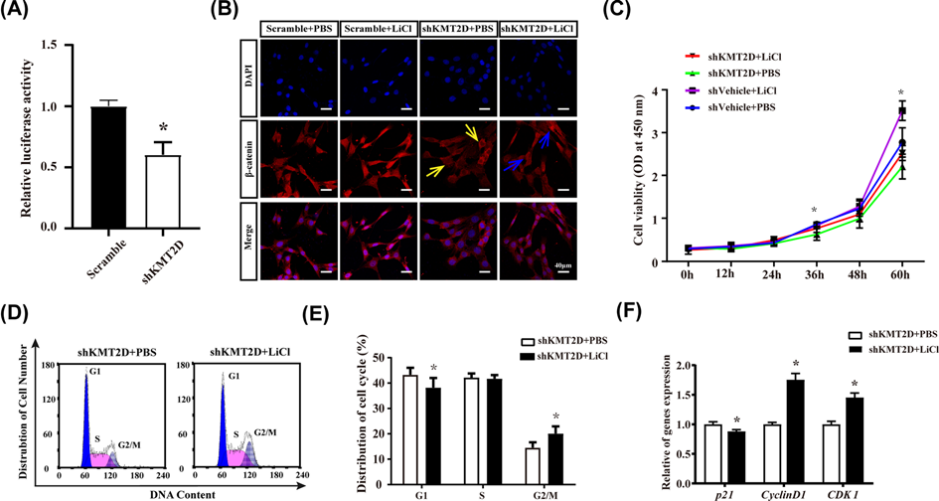
LiCl partially reversed the effects of KMT2D deficiency in the homeostasis of cell proliferation and cell cycle (**A**) Luciferase assay was performed to evaluate the reduction in Wnt/β-catenin signaling activity (TOP/FOP) induced by KMT2D knockdown. (**B**) KMT2D knockdown resulted in the decrease in nuclear β-catenin (yellow arrows). LiCl stimulation partially reversed the decrease in nuclear β-catenin induced by KMT2D knockdown (blue arrows). (**C**) Result of CCK8 assay showed that the cell proliferation reduction in shKMT2D group was reversed by LiCl stimulation. OD values at 450 nm spectrum of three independent experiments were presented. (**D**) LiCl (5 mM) alleviated the G_1_ phase arrest after KMT2D knocking down. (**E**) Quantitative data of cell cycle distribution showed G_1_ phase arrest released by LiCl. (**F**) CyclinD1 and Cdk1 were up-regulated by LiCl, and p21 was down-regulated by LiCl. The data represent three independent experiments, each in triplicate. Each bar represents mean ± SD from three samples (*P<0.05). Means were compared using Student’s *t* test between two groups (A,E,F). A normality test was run before one-way ANOVA (C).

TOP/FOP assay result showed that Wnt/β-catenin activity decreased after KMT2D knockdown (Figure 5A). Then KMT2D knockdown cells were stimulated with the Wnt signaling agonist LiCl (5 mM in PBS) to further explore the role of the Wnt signaling pathway in the regulation of KMT2D. Cell immunofluorescence assays showed that KMT2D knockdown decreased nuclear β-catenin, and LiCl stimulation partially reversed the reduction in nuclear β-catenin (Figure 5B). CCK8 assays revealed that LiCl partially reversed the reduction in the cell proliferation activity caused by KMT2D knockdown (Figure 5C). Furthermore, flow cytometry results indicated that LiCl, to some extent, alleviated the G_1_ phase arrest induced by KMT2D knockdown (Figure 5D,E). With LiCl stimulation for 24 h, the expression of *cyclinD1* and *Cdk1* was up-regulated, whereas that of *p21* was slightly down-regulated, which might be responsible for the G_1_ phase release (Figure 5F).

**Figure 6:**
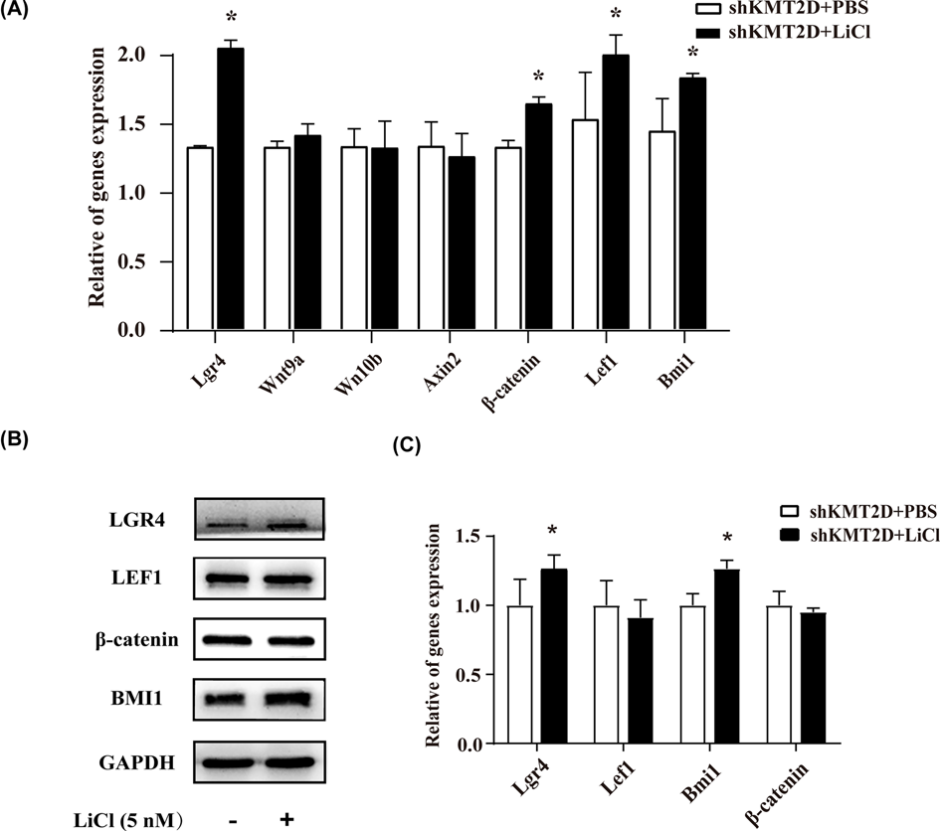
LiCl partially up-regulated the expressions of Kmt2d-target Wnt factors in Kmt2d knockdown cells (**A**) The mRNA expression of Lgr4, β-catenin, Lef1 and Bmi1 were significantly increased after the stimulation of LiCl. (**B**) Lgr4 and Bmi1 were up-regulated at protein level after the stimulation of LiCl by Western blot, Lef1 and β-catenin showed no change. (**C**) Quantitative data of Western blot of (B). The data represent three independent experiments, each in triplicate. Each bar represents mean ± SD from three samples (*P<0.05). Means were compared using Student’s *t* test between two groups (C). A normality test was run before one-way ANOVA (A).

In *Kmt2d* knockdown cells, the expressions of *Kmt2d*-target Wnt factors were compared between the shKMT2D+PBS and shKMT2D+LiCl group, in order to figure out whether or not LiCl stimulation may change the target gene expressions. As earlier referred in the Figure 4A, the mRNA expression levels of *Lgr4, Wnt9a, Wnt10b, Axin2, Lef1* and *β-catenin* were decreased in KMT2D knockdown cells. After LiCl stimulation on KMT2D knockdown cells, the mRNA levels of *Lgr4*, *β-catenin*, *Lef1* and *Bmi1* were partially rescued, whereas *Wnt9a* and *Wnt10b* showed no obvious changes (Figure 6A). Protein expression of LGR4, a Wnt signaling receptor, was up-regulated in KMT2D knockdown cells after LiCl stimulation. Simultaneously, LiCl stimulation up-regulated the protein level of BMI1 (Figure 6B,C). The results suggested that Wnt/β-catenin signaling pathway might be involved in the regulation of KMT2D in dental epithelial cell line proliferation and cell cycle homeostasis.

## Discussion

It is important to elucidate the genetic and epigenetic mechanisms underlying dental epithelium proliferation to understand the process of early tooth development. The H3K4 methyltransferase KMT2D plays a vital role in regulating the development of multiple organs, including adipogenesis and myogenesis [21], cardiac development [26], B-cell development [27,28], epidermal homeostasis [22] and craniofacial development [29]. Recently, *Kmt2d* mRNA has been observed in the developing osteoblasts, epithelia and neural tissues [30]. During tooth development, Porntaveetus et al. detected *Kmt2d* mRNA in the bud and cap stages of human dental epithelial tissues using *in-situ* hybridization [19]. Nevertheless, a systematic exploration of KMT2D and H3K4me patterns in the process of tooth development is still needed for understanding the specific gene function. In the present study, we systematically analyzed the expression pattern of KMT2D in different stages of mouse molar development. The results showed that KMT2D protein was mainly expressed in the enamel organ from bud stage to early bell stage, and the expression pattern was similar to that of the cell proliferation marker Ki67 and cell cycle marker pRb. The expression of pRb is closely associated with G_1_–S transition, yet the pRb protein has also been reported to function in other fields including tooth differentiation. et al. have reported that Rb1 mRNA appears upregulate-d in differentiating ameloblasts and odontoblasts, suggesting roles for Rb1 in tooth differentiation [31]. The specific expression of KMT2D in dental epithelial cells of early tooth germ suggests that KMT2D might function in the initiation of tooth development and enamel organ formation. Moreover, we found that KMT2D was expressed in both ameloblast and odontoblast layers at the late bell stage, which suggested that KMT2D might as well play a role in late stages of tooth development.

LS8, a cell line derived from mouse enamel organ epithelial, was used for investigate the function and mechanism of KMT2D in dental epithelial cells [32]. Results showed that KMT2D knockdown significantly reduced the proliferation activity and self-renewal capacity of dental epithelial cell line. Besides, two other shRNAs targeting KMT2D have been designed and confirmed to influence LS8 cell viability, which suggested that the effect of KMT2D on cell proliferation was not due to off-target effects (Supplementary Figure S1). We further found that KMT2D knockdown resulted in reduction in cell cycling activity in dental epithelial cell line, which might be one reason of the cell proliferation reduction. Cyclin D1, as an important cell cycle regulator in the transition from G_1_ to S phase [33], its expression was significantly reduced after KMT2D knockdown. Consistent with KMT2D effect in dental epithelial cell line, several studies have reported the function of KMT2D in cell proliferation and cell cycle. Lin-Shiao et al*.* had reported that the function of KMT2D in cell proliferation regulation of normal epidermal cells was consistent with our result in dental epithelial cell line [22]. During mouse heart development could cause G_0_/G_1_/S arrest of myocardial cells, which explains the phenomenon of myocardial hypoplasia [26]. However, the role of KMT2D in craniofacial epithelial cell line LS8 proliferation and cell cycle homeostasis is first reported in our study. It needs to be clarified that the dental epithelial cell line LS8 used in this experiment has been transformed, its biological characteristics are different from that of primary dental epithelial cells. Although the LS8 cell line is widely used in dental epithelial research, it cannot represent the performance of developing dental epithelial cells. KMT2D is a major causative gene of Kabuki syndrome with teeth dysplasia including missing teeth and conical teeth [17,18]. Loss-of-function KMT2D variants have been reported in Kabuki patients [15,34]. In consideration of our results, the abnormal dental phenotype in Kabuki patients might be associated with the disturbance of dental epithelial cell proliferation that caused by KMT2D deficiency. Recently, Shpargel et al. found that knocking out KMT2D in mouse neural crest would lead to abnormal craniofacial development, yet no teeth deformities was reported [35]. This phenomenon might be due to the redundant function with KMT2C homolog or UTX cofactor, as has been observed in other cell types [20,36]. As a major mammalian H3K4 methyltransferase, KMT2D is considered to be essential for enhancer activation by altering the level of H3K4me [28,37]. In KMT2D knockdown cells, we found significant decreases in total H3K4me1 and H3K4me3 levels in nuclear proteins, corroborating the function of KMT2D in H3K4me regulation.

During embryo development and tissue formation, KMT2D is a critical factor during classical signaling pathways regulation. In the present study, RNA-seq and bioinformatics analysis was carried out in the KMT2D deficient dental epithelial cell line, and several important pathways in early tooth development were screened out to be significantly affected by KMT2D knockdown, including Wnt, Notch and Hedgehog pathways. Particularly, eight Wnt signaling pathway factors including *Lgr4*, *Wnt9a*, *Wnt10b*, *Tle2*, *Tle6*, *Axin2*, *Lef1* and *β-catenin* were confirmed to be down-regulated after KMT2D knockdown. During odontogenesis, classical Wnt/β-catenin signaling is generally considered to function in cell proliferation and differentiation [38,39]. Tight regulation of the Wnt/β-catenin pathway in dental epithelium has been proven to be essential for tooth patterning in early tooth formation. Abnormal activation of Wnt/β-catenin signaling led to continuous tooth generation in mice [5]. Combined with our results, it might be conjectured that the abnormal tooth number and morphogenesis observed in Kabuki patients would be attributed to the effects of KMT2D deficiency on Wnt signaling. *Bmi1*, as an activator of Wnt signaling [40], has been shown to regulate self-renewal activity and cell proliferation [41–43]. Our results demonstrated that *Bmi1* was also down-regulated upon KMT2D knockdown, and was partially rescued by the activation of Wnt/β-catenin signaling. These results further indicate that KMT2D might be closely related with Bmi1 regulation and the self-renewal processes of dental epithelial cell line.

To clarify the relationship of KMT2D and Wnt signaling in dental epithelial cell line behaviors, the rescue experiments were performed using LiCl, a classic Wnt signaling pathway activator. The results showed that LiCl partially reversed the effects of KMT2D knockdown on the cell proliferation and cell cycle, suggesting that Wnt/β-catenin signaling is involved in KMT2D regulation of epithelial cell proliferation activity. As one of the important Wnt signaling pathway receptors, Lgr4 (leucine-rich repeat-containing GPCR 4) has been reported to be expressed in cervical loop epithelium during embryonic tooth morphogenesis [44]. Zhou et al*.* had demonstrated that Lgr4 knockdown affected the proliferation of dental papilla stem cells and reduced the amount of β-catenin located in nuclei [45]. In our study, Lgr4 was down-regulated and the nuclear β-catenin was decreased in KMT2D knockdown cells, while LiCl stimulation partially increased the Lgr4 expression and nuclear β-catenin level. From these results, it can be speculated that Lgr4 might be involved in the regulation of dental epithelial cell line proliferation by affecting β-catenin nuclear translocation. Further research is needed to be carried out to explore the precise mechanism and the key target factor of Wnt signaling pathway involved in KMT2D regulation of LS8 cell proliferation. Compared with BIO (6-bromo-indirubin-3′-oxime) and Wnt protein ligands, LiCl is a broad-spectrum agent. Apart from activating the canonical Wnt signaling pathway activity by inhibiting GSK-3β, LiCl could also activate adenylyl cyclase, IP3, PKC and arachidonic acid signaling [46]. These non-specific effects of LiCl might be one reason that the rescue levels of many Wnt markers seem to be modest in our assays. It is noteworthy that Wnt signaling stimulation could only partially reverse the cell proliferation level, which might be caused by some redundant effect of other possible KMT2D downstream factors (Shh and Notch), or the low LiCl concentration (5 nM) that we selected according to LS8 cell state. Nevertheless, our *in vitro* experiments shed new light on the function of KMT2D and H3K4me in dental epithelial cell line, and provide basis for further *in vivo* studies using conditional transgenic mice.

## Conclusion

In conclusion, the present study elucidated that KMT2D presents a specific spatiotemporal expression pattern during tooth germ development, and regulates cell proliferation and the cell cycle of dental epithelial cell line partially via Wnt/β-catenin signaling. Our study provides new insights into the role of KMT2D and histone methylation in dental development and furthers our understanding of the molecular pathogenesis of Kabuki syndrome.

## Supplementary Material

Supplementary Figure S1

## Data Availability

The datasets used or analyzed during the current study are available from the corresponding authors on reasonable request.

## Abbreviations

Cdk: cyclin-dependent kinase
DEG: differential expression gene
DKK1: Dickkopf 1
EdU: 5-ethynyl-2'-deoxyuridine
GFP: green fluorescent protein
GO: Gene Ontology
H3K4me: histone 3 lysine 4 methylation
H3K4me1: histone 3 lysine 4 monomethylation
H3K4me3: histone 3 lysine 4 trimethylation
KEGG: Kyoto Encyclopedia of Genes and Genomes
KMT2D: lysine methyltransferase 2D
Lgr4: leucine-rich repeat-containing GPCR 4
LiCl: lithium chloride
MOI: multiplicity of infection
pRb: phosphorylation retinoblastoma protein, Ser^780^
RNA-seq: RNA-sequencing
RT: room temperature
RT-qPCR: quantitative reverse transcription polymerase chain reaction
