# Supplementary material for: KMT2D deficiency disturbs the proliferation and cell cycle activity of dental epithelial cell line (LS8) partially via Wnt signaling
Source: Biosci Rep. 2021 Nov 18;41(11):BSR20211148. doi: 10.1042/BSR20211148 (PMC8607332; doi:10.1042/BSR20211148)

Supplemental figure 1. CCK8 results showed that shKMT2D-1(sh1426) and shKMT2D-3(sh6945) showed that KMT2D knockdown influenced LS8 cell viability. (a) The knockdown efficiency of KMT2D was confirmed by Western blot. (b) Knockdown KMT2D with shKMT2D-1(sh1426) resulted lower viability in LS8 cell line. (c) Knockdown KMT2D with shKMT2D-3(sh6945) resulted lower viability in LS8 cell line.

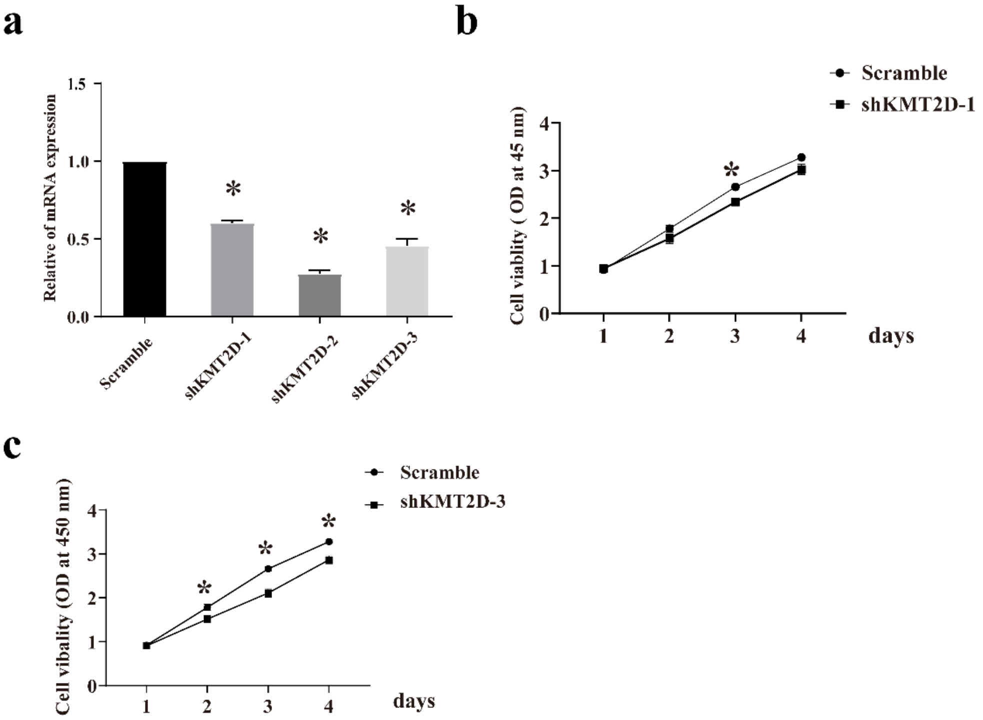

Supplement: Supplementary Figure S1 [file BSR-2021-1148_supp.pdf]
